# *In vitro* Evaluation of Anthelmintic Activity of *Nauclea orientalis* Leaves

**DOI:** 10.4103/0250-474X.73932

**Published:** 2010

**Authors:** S. T. V. Raghavamma, N. Rama Rao

**Affiliations:** Chalapathi Institute of Pharmaceutical Sciences, Chalapathi Nagar, Lam, Guntur-522 034, India

**Keywords:** Anthelmintic activity, *nauclea orientalis*, paralysis and death, pheritima postuma

## Abstract

Antianthelmintic activity of successive extracts (chloroform, acetone, ethanol and aqueous) of *Nauclea orientalis* leaves were evaluated separately on adult Indian earthworm (*Pheretima posthuma*) and compared with that of albendazole. It was found that the extracts exhibited, respectively dose-dependent action and inhibition of spontaneous motility (paralysis) and death of earthworms. The results indicated that the chloroform, ethyl acetate and ethanol extracts were more potent.

Helminthic infestations are now being recognized as a cause of chronic ill health and sluggishness amongst the children. More than half of the population in the world suffers from worm infestations of one or the other. Helminthes also affect domestic animals and livestock causing considerable economic loss. Traditional system of medicine reports the efficacy of several natural products eliminating helminthes[1]. Considering this *Nauclea orientalis* has been evaluated for its anthelminthic activities.

*Nauclea orientalis* (Family: Rubiaceae) is a large tree distributed in India and is indigenous to north Australia[2]. The concoction from seed pulp is used to cure cough, cold, stomach pain, vomiting and diarrhea[2]. Literature review showed the presence of indole alkaloidal glycosides and nine angustine-type alkaloids in the ammoniacal extracts of leaf. Three of them are 10-hydroxyangustine and the two diastereoisomeric 3,14-dihydroangustolines. These compounds were found to exhibit *In vitro* antiproliferative activity against the human breast carcinoma MCF-7 and murine leukemic cell lines[3]. The anthelminthic activity of the plant extract has not been reported in the literature. In the present study, anthelmintic potential of different extracts of leaf have been evaluated.

The leaves of *N. orientalis* were collected from Lam, Guntur during June/July 2007. The plant was identified and authenticated by the Department of Botony, Acharya Nagarjuna University, Nagarjuna Nagar. The leaves were cleaned; shade dried, coarsely powdered and sieved using # 40. The powder was extracted in a Soxhlet apparatus using petroleum ether, chloroform, ethanol and distilled water as solvents. Extracts were subjected to Rotary vacuum evaporation[4]. Various standard phytochemical tests were performed on the extracts to identify the active chemical constituents[5,6].

The anthelmintic activity was evaluated on adult Indian earthworms, *Pheritima postuma* (Annelida), due to its anatomical and physiological resemblance with intestinal round worm parasites of human beings[7–10]. The worms were collected and identified at Vermi compost Division, Regional Agricultural Research Institute, Lam, Guntur.

The anthelmintic method was carried as per the method of Pal *et al*.,[11], with minor modifications. Sixteen groups each containing six earthworms of approximately equal size were released into 10 ml of desired formulation. Each group was treated with, albendazole, chloroform extract, ethyl acetate extract, ethanol extract and aqueous extract (40, 60 and 80 mg/ml) in normal saline with 5% DMF and in vehicle alone acting as control. Time for paralysis was noted when no movement could be observed with a slight pin prick method. Time for death of individual earth worms was recorded when the worms showed no movement either by vigorous shaking or by dipping in warm water.

Preliminary phytochemical screening of chloroform and ethyl acetate extracts showed the presence of tannins, sterols and flavanoids while ethanol extract showed the presence of tannins, glycosides, phenols and saponins. Data in the Table 1, revealed that all the extracts of *N. orientalis* leaves possessed dose dependent and significant anthelmintic activity when compared to that exhibited by albendazole on earthworm. Among all the extracts, ethanol extract required least time to cause paralysis. Chloroform and ethyl acetate extracts showed minimum death time. Aqueous extract had shown to posses least activity than all the extracts. The time required for causing paralysis (P) in case of ethanol extract is 3 min and death (D) in 15 min, while chloroform extract showed P and D in 4.0 min and 11.2 min, respectively. Finally ethyl acetate showed P and D in 7.3 min and 11.3 min. Albendazole showed 1.2 min and 5.2 min for the same observations.

**TABLE 1:** ANTHELMINTIC ACTIVITY OF CHLOROFORM, ETHYL ACETATE AND ETHANOL LEAVF EXTRACTS OF *NAUCLEA ORIENTALIS*

Test Subs	Concentration (mg/ml)	Time taken for paralysis (P) and death (D) (min)	
		*P. posthuma*	
		P	D
Albendazole	40	2.20±0.05	7.49±0.03
	60	1.30±0.01	6.24±0.05
	80	1.25±0.02	5.25±0.06
Chloroform extract	40	6.45±0.06	11.45±0.06
	60	5.30±0.01	11.25±0.03
	80	4.00±0.02	11.20±0.05
Ethyl acetate extract	40	8.28±0.01	14.00±0.6
	60	7.00±0.02	13.00±0.5
	80	7.30±0.04	11.30±0.06
Ethanol extract	40	5.00±0.03	18.10±0.8
	60	4.20±0.05	17.70±1.12
	80	3.00±0.27	15.50±0.05
Aqueous extract	40	15.00±0.05	183±2.25
	60	17.00±0.01	150±3.29
	80	16.00±0.05	122±1.32
Control	-	-	-

Results are expressed as mean ±SEM of three observations

P- Paralysis

D- Death

The predominant action of albendazole on worm is inhibitory action on microtubular function. The leaf extracts not only showed paralysis but death of the organism with increase in concentrations. Phytochemical analysis of the crude extracts showed the presence of tannins and saponins as one of the chemical constituents. Tannins and saponins were shown to possess anthelminthic activity[12,13]. Tannins are found to bind to free proteins in the gastrointestinal tract of the host animal or glycoprotein on the cuticle of the parasite and cause death[14].

In conclusion, only the anthelmintic activity was evaluated for the leaf extracts of *N. orientalis*. The crude extracts of *Nauclea orientalis* has to be further studied to isolate the active compounds present and to establish the mechanism of action.
